# An Interfacial Affinity Interaction-Based Method for Detecting HOTAIR lncRNA in Cancer Plasma Samples

**DOI:** 10.3390/bios12050287

**Published:** 2022-04-28

**Authors:** Kimberley Clack, Narshone Soda, Surasak Kasetsirikul, Richard Kline, Carlos Salomon, Muhammad J. A. Shiddiky

**Affiliations:** 1 School of Environment and Science, Nathan Campus, Griffith University, Nathan, QLD 4111, Australia; kimberley.clack@griffithuni.edu.au; 2 Queensland Micro and Nanotechnology Centre, Nathan Campus, Griffith University, Nathan, QLD 4111, Australia; surasak.kasetsirikul@griffithuni.edu.au; 3 School of Engineering and Built Environment (EBE), Nathan Campus, Griffith University, Nathan, QLD 4111, Australia; 4 Section of Gynaecologic Oncology, Ochsner Clinic Foundation, New Orleans, LA 70121, USA; rkline@ochsner.org (R.K.); c.salomongallo@uq.edu.au (C.S.); 5 Exosome Biology Laboratory, Centre for Clinical Diagnostics, University of Queensland Centre for Clinical Research, Royal Brisbane and Women’s Hospital, The University of Queensland, Brisbane, QLD 4029, Australia; 6 Departamento de Investigación, Postgrado y Educación Continua (DIPEC), Facultad de Ciencias de la Salud, Universidad del Alba, Santiago 8320000, Chile

**Keywords:** HOTAIR lncRNA, interfacial biosensing, affinity interaction, RNA biosensor, electrochemical detection, ovarian cancer

## Abstract

Long non-coding RNA Homeobox transcript antisense intergenic RNA (HOTAIR) is recognized as a participant in different processes of normal cell development. Aberrant overexpression of HOTAIR contributes to the initiation, growth, and invasiveness of ovarian cancer. Using the affinity interaction of target HOTAIR lncRNA sequences towards a screen-printed gold electrode (SPE-Au), herein we report on a novel, rapid and simple method to detect HOTAIR sequences. HOTAIR lncRNA sequences were first extracted from ovarian cancer cell lines and patient plasma samples and were magnetically captured and purified by complimentary capture probe-functionalized magnetic beads. Isolated target HOTAIR lncRNAs were directly adsorbed onto unmodified screen-printed gold electrodes (SPE-Au) for direct quantification with [Fe(CN)_6_]^3−/4−^ redox couple. Our assay achieved a linear dynamic range of 100 nM and 1 pM for detecting pre-clinical model HOTAIR lncRNA samples (%RSD ≤ 5%, for *n* = 3) and was highly specific, showing clear distinction between HOTAIR lncRNA targets and non-specific miR-891 and miR-486 (100 nM) (%RSD ≤ 5%, for *n* = 3). The method was tested using ovarian cancer-specific cell lines (SKOV3 and OVCAR3) and mesothelial cell line (MeT-5A)-derived lncRNAs. The analytical performance of our method was validated using RT-qPCR. Finally, the method was tested using clinical samples from ovarian cancer patients and the resulting electrochemical responses show a clear distinction between the ovarian carcinoma and benign samples.

## 1. Introduction

Long non-coding RNAs (lncRNAs) represent a subset of regulatory ncRNAs that are recognized as key regulators of physiology and pathology, being involved in processes such as epigenomic modulation, the regulation of post-transcriptional gene expression, and gene remodeling [1,2]. HOX antisense intergenic RNA (HOTAIR)—a crucial lncRNA—has been shown to participate in different processes of normal cell development [3,4]. The aberrant expression of long non-coding HOTAIR has been linked to many cancers, such as ovarian, colorectal, pancreatic, primary breast and prostate [5,6,7]. HOTAIR upregulation has been shown to increase the proliferation, migration, and invasiveness of ovarian cancer cells, while the inhibition of HOTAIR represses cell viability and promotes apoptosis [8]. As a result, the early detection of HOTAIR lncRNA has emerged as a promising strategy for the early detection of cancer.

Several molecular biology-based approaches have been developed over the years for the analysis of HOTAIR and other lncRNA sequences. Such approaches include microarrays, Northern blotting, next-generation sequencing and RT-qPCR [9,10]. While these approaches have their merits, they suffer from limitations such as high equipment cost, lower specificity, and reduced dynamic range, being time and labor intensive, and they may not be suitable for high-throughput analysis [11,12]. Furthermore, RT-qPCR fluorescent labels are susceptible to background fluorescence interference, as well as amplification bias [13]. As such, a relatively simple, cheap, sensitive, and rapid method of HOTAIR detection could provide a better route for the routine monitoring of ovarian cancer. However, the overarching challenge of HOTAIR lncRNA biomarker detection is that it is often present in bodily fluids in very low concentrations (usually several nanograms per milliliter), and due to the varying expression levels of HOTAIR, a biosensing strategy with a broad dynamic range is required [14,15].

Electrochemical assays/biosensors have shown great promise for alleviating the above issues, due to their high sensitivity and specificity, low cost and miniaturization potential [16]. Recent advances have suggested that the affinity interaction between a bare metal surface (i.e., gold, platinum, etc.) and biological molecules (e.g., DNA, RNA, and proteins) depends on the molecular structures and three-dimensional conformations of the adsorbents, and follows conventional physisorption and chemisorption mechanisms [17,18]. Direct quantification of the biomolecule is achieved based on the interaction, or the affinity of the biomolecule with the bare metal surface. To date, interfacial biosensing has been used to detect nucleic acid sequences, small molecules, DNA methylation and mutations, proteins [19,20,21,22] etc. The adsorption of DNA onto metal surfaces has been well studied, with the intermolecular forces behind the DNA–gold adsorption phenomenon being attributed to electrostatic interactions, hydrophobic forces, and specific DNA–gold bonding [23,24]. Thus, this affinity interaction is the basis of the current method—a method by which one could directly adsorb pre-isolated HOTAIR targets onto a bare gold electrode and quantify the adsorbed HOTAIR targets via electrochemical methods. The use of the direct adsorption of target HOTAIR on a bare gold electrode rather than the conventional hybridization method of using recognition and transduction layers in nucleic acid biosensors is a significant improvement in biosensing design as it substantially simplifies the method by avoiding the hybridization steps and other complicated chemistries underlying each step of the fabrication [23]. Interfacial biosensing also provides significant advantages such as rapidness and being inexpensive; however, the approach requires highly purified samples for avoiding signal interference from non-specific biomolecules [18].

To further improve assay sensitivity, biosensors often utilize the unique multifunctional properties of nanomaterials to facilitate HOTAIR lncRNA target purification by featuring a high surface area and porosity, excellent biocompatibility and magnetic properties [25]. These properties of nanoparticles may serve as anchoring sites for biorecognition components such as antibodies, enzymes, single-stranded RNA (ssRNA) or single-stranded DNA DNA (ssDNA) [25]. These biorecognition components allow the HOTAIR lncRNA targets to be magnetically captured, purified, and isolated before being brought directly to the sensor surface, greatly reducing the availability of non-HOTAIR targets that would otherwise cause biofouling at the sensor surface. This biosensing strategy/miniaturization platform offers rapid detection and low-cost analysis, eliminating the need for amplification, target labelling, and sensor surface functionalization [26].

**Figure 1:**
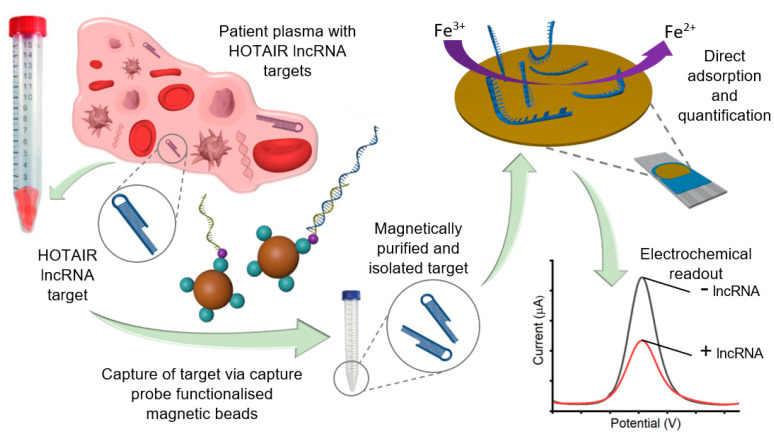
Schematic representation of the detection of HOTAIR lncRNA with the [Fe(CN)_6_]^3−/4−^ redox system for DPV readout. Magnetic nanoparticles functionalized with complimentary capture probe sequences were added to total extracted RNA samples containing HOTAIR lncRNA targets. After hybridization of HOTAIR targets and capture probes, the capture probe/nanoparticle/target complex was magnetically washed for HOTAIR target purification. HOTAIR lncRNA targets were isolated from the capture probe/nanoparticle complexes via heat release denaturation. HOTAIR targets were then collected and directly adsorbed at the unmodified SPE-Au surface for 30 min via the gold-RNA affinity interaction, before voltametric interrogation with [Fe(CN)_6_]^3−/4−^ redox couple for generating the DPV readout.

Herein, we demonstrate the further utility of our previously developed method for a simple and amplification-free approach for early detection of HOTAIR lncRNA pre-isolated from patient plasma using the interfacial affinity interaction between gold and target HOTAIR lncRNAs (Figure 1). Unlike our previously reported method, which exploited the long poly-A tail sequences contained within messenger RNA, this approach combines three subsequent steps: (i) initial extraction of total RNA from clinical samples; (ii) magnetic nanoparticle-mediated capture, pre-concentration, and heat release of the target HOTAIR lncRNA from the total RNAs mixture; and (iii) subsequent detection of isolated HOTAIR lncRNA using the interfacial affinity interaction. We demonstrate detection of as low as 1 pM from samples containing designated proportions of synthetic HOTAIR targets spiked in buffer and healthy plasma samples. We also demonstrate the successful analysis of lncRNAs extracted from ovarian carcinoma cell lines and patient plasma samples. Finally, our method has been validated with the RT-qPCR method.

## 2. Materials and Methods

### 2.1. Reagents and Materials

Unless otherwise stated, chemicals and reagents were used without further purification and were of analytical grade. Screen-printed gold electrodes (SPE-Au) with a three-electrode system (DRP-220AT) were purchased from Metrohm Dropsens (Spain). In the three-electrode system, working (4 mm diameter), reference and counter electrodes consisted of gold, silver/silver chloride, and platinum, respectively. UltraPure^TM^ DNase/RNase-free distilled water was purchased from Invitrogen, Australia. Synthetic lncRNA and capture probes (Table 1) were purchased from Integrated DNA Technologies (Coralville, IA, USA). All other reagents were purchased from Sigma Aldrich (Sydney, NSW, Australia). All electrochemical measurements were performed using a 650E series electrochemical workstation potentiostat purchased from CH Instruments, Austin, TX, USA. Plasma samples (two benign and four ovarian carcinomas) were obtained from UQCCR according to the declaration of Helsinki, with approval from the Ethics Committee of the University of Queensland (approval number 2016000300) and the Ochsner Medical centre (New Orleans, LA, USA). MeT-5A, SKOV3, and OVCAR3 cell lines were cultured in the laboratory.

### 2.2. Preparation of RNA from Cell Line and Ovarian Cancer Samples

Cells (MeT-5A, SKOV3, and OVCAR3) were cultured in 25 cm^2^ flasks with RPMI-1640 growth medium (Life Technologies, Melbourne, Australia) containing 1% penicillin/streptomycin (Life Technologies, Australia) supplemented with 10% fetal bovine serum (Life Technologies, Australia) with humidification at 5% CO_2_ and 37 °C. A standard trypsinization protocol was used to harvest the cells once they had reached 70% confluence. The cells were washed with 2 mL of HBBS buffer solution (Gibco) to remove enzyme inhibitors, and 2 mL of TRyPLe cell dissociation enzyme (Gibco) was added for incubation at 37 °C for three min. Trypsin activity was neutralized with 2 mL of cell culture media and centrifugation for 5 min at 2500 RPM. Once the cell pellet had been obtained, total RNA was extracted from cancer cell lines (MET-5A and OVCAR-3) using an RNeasy mini kit 50 (Qiagen, Victoria, Australia) as per manufacturer recommendations.

### 2.3. Magnetic Isolation and Purification of HOTAIR lncRNA Target

In brief, extracted total RNAs were hybridized with complementary biotinylated capture probe (Table 1) sequences by incubation on the thermomixer for one hour at 25 °C at 300 rpm. After the incubation of the capture probe and HOTAIR target sequences, previously washed magnetic beads were added to the hybridized solution. Thermomixing at room temperature at 300 rpm for 30 min facilitated the probe to nanoparticle binding via the streptavidin-biotin affinity interaction. Nanoparticle/HOTAIR target complexes were magnetically washed with 2 × binding and wash buffer to purify the HOTAIR target sequences, followed by resuspension in RNAse-free water. HOTAIR targets were denatured from the capture probe sequences via a 2 min heat release at 95 °C. The released HOTAIR targets were diluted with 5 × SSC buffer (pH 7.0) in preparation for electrochemical quantification.

### 2.4. Electrochemical Detection of HOTAIR lncRNAs

Isolated target HOTAIR lncRNAs were directly adsorbed at the surface of chemically cleaned screen-printed electrodes for a 30 min incubation time. Unbound species were rinsed away (3 × washing) with 10 mM phosphate-buffered saline (PBS) solution. A 2 mM ferricyanide/ ferrocyanide redox couple solution was added to the SPE-Au surface, and differential pulse voltammetry (DPV) (initial voltage −0.15 V; final voltage 0.35 V; amplitude 0.05 V) measurements were carried out to quantify the amount of HOTAIR lncRNA adsorbed at the sensor surface.

### 2.5. Quantitative Reverse-Transcription Polymerase Chain Reaction (RT-qPCR)

Synthetic primer sequences for amplifying HOTAIR (for RT-qPCR) and GAPDH (housekeeping gene) were designed (Table 1). cDNA synthesis was performed using a miScript Reverse Transcription kit (Qiagen, Germany). To verify the expression of HOTAIR, each qPCR reaction was performed in a total volume of 20 µL containing approximately 100–150 ng, of cDNA template and 10 μM each of forward and reverse primers. 2XSensiMix SYBR No-ROX master mix (Bioline) was used for RT-qPCR analysis. qPCR was performed on a CFX96 (Bio-Rad) thermocycler with the following conditions: thermal cycling was initiated with an initial denaturation step at 95 °C for 10 min followed by 40 cycles of 95 °C for 15 s (denaturation), 55 °C for 15 s (annealing), and 72 °C for 15 s (extension). A no-template control (NoT) was included in the PCR assays and all samples were performed in triplicate.

## 3. Results and Discussion

### 3.1. Assay Principle

**Figure 2:**
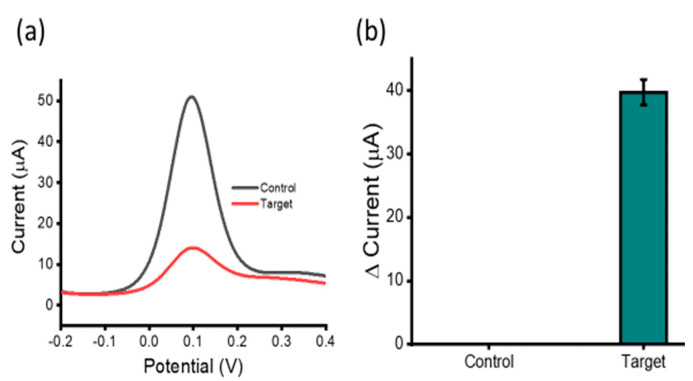
Assay functionality: Differential pulse voltammetry (DPV) readout depicting current responses for 100 nM synthetic HOTAIR lncRNA target and NoT (**a**,**b**) illustrating the change in current observed between the HOTAIR targets and control. Error bars represent the relative standard deviation across three repeated experiments.

Figure 1 illustrates the assay principle of HOTAIR lncRNA detection. HOTAIR lncRNA extracted from plasma samples was magnetically captured, purified, and isolated via heat denature, for direct adsorption onto an SPE-Au. Subsequent electrochemical detection was performed using voltametric interrogation with the [Fe(CN)_6_]^3−/4−^ redox couple for DPV readout and quantification. Briefly, biotinylated capture probe sequences (complementary to HOTAIR lncRNA targets) bound to streptavidin-coated magnetic beads were allowed to hybridize with HOTAIR lncRNA targets in solution via the biotin–avidin interaction. Captured HOTAIR lncRNA targets were then magnetically purified through several washing steps, allowing the removal of non-targets from the solution, leaving behind the nanoparticle/capture probe/HOTAIR target complexes. A heat release step was performed to denature the HOTAIR targets from the capture probe/nanoparticle complexes, for removal from the solution. Released HOTAIR targets were then directly adsorbed on the bare, unmodified metal interface via the gold-RNA affinity interaction for subsequent electrochemical detection. To evaluate the assay functionality, we investigated our assay in the presence and absence of 100 nM synthetic HOTAIR lncRNA (Figure 2a). Previous reports have demonstrated that [Fe(CN)_6_]^3−/4−^ redox system alone can be utilized to quantify surface bound nucleic acids on a gold surface [27,28]. Electrochemical detection yielded almost four-fold reduced current response in comparison to the control, which demonstrates the functionality of our assay (Figure 2b). When adsorbed on the gold sensor surface via the RNA–gold affinity interaction, the negative charges experience coulombic repulsion between ferricyanide ions. This repulsion results in a decreased faradaic current signal (for the one-electron transfer reaction) compared to the control [13].

### 3.2. Analytical Performance of the Assay

**Figure 3:**
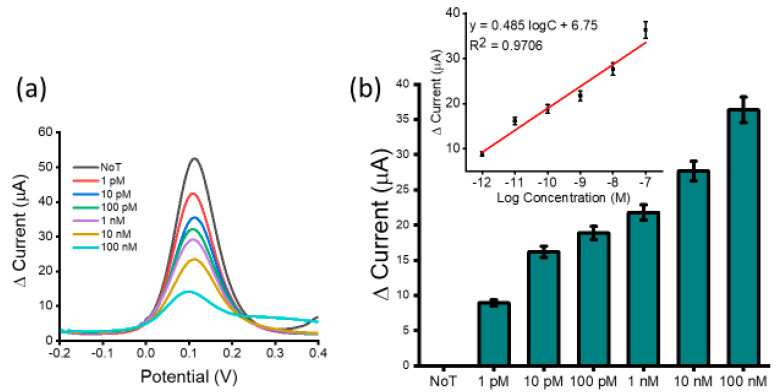
Assay sensitivity: differential pulse voltammetry (DPV) readout depicting current responses for varying concentrations of synthetic HOTAIR lncRNA (**a**) and relative DPV signal change for various synthetic HOTAIR lncRNA concentrations with respect to the NoT (**b**). Error bars represent the standard deviation across three separate experiments. Inset shows the analogous linear calibration plot for current change in reference to the log concentration.

To determine assay detection sensitivity, a serial dilution of synthetic HOTAIR lncRNA targets was performed and detected (Figure 3a). We observed a decrease in DPV signal current with an increase in HOTAIR lncRNA concentration. This was due to higher amounts of HOTAIR lncRNA target blocking the sensor surface. A higher amount of adsorbed HOTAIR lncRNA caused a higher coulombic repulsion of the approaching [Fe(CN)_6_]^3−/4−^ ions, hindering their diffusion at the electrode surface, resulting in a lower Faradaic current. Detection sensitivity was tested within the range of 100 nM and 1 pM and the calibration plot showed good linearity. The linear regression equation was estimated to be *y* = 0.485 logC + 6.75 (*y* is the change in current and *x* concentration of HOTAIR), and a correlation coefficient of *R*^2^ = 0.9706 (Figure 3b inset). In comparison to the NoT (no-template control which features an unblocked sensor surface generating a high current reading), the assay could detect up to 1 pM of target HOTAIR lncRNA with reproducibility of % RSD ≤ 5%, for *n* = 3. This showed that the sensor was able to detect surface blocking attributed to the presence of a low concentration of the target HOTAIR lncRNA. The performance of several similar biosensors for lncRNA was investigated (Table 2). The analysis showed that our developed assay was comparable to previously reported assays for lncRNA with respect to the detection limit and linear range. However, our assay is advantageous concerning simplicity and cost. Furthermore, it does not require electrode surface functionalization, target amplification/extension, labelling, or the use of antibodies.

**Figure 4:**
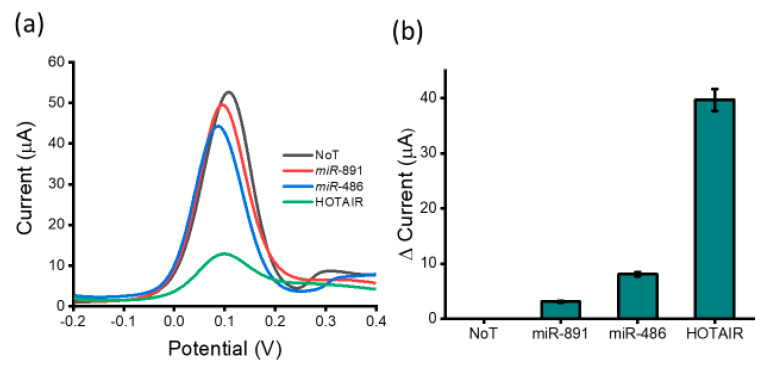
Assay specificity: (**a**) DPV readout displaying current output after the assay was tested with various miRNA sequences (miR-891 and miR-486 (100 nM), NoT and target HOTAIR lncRNA (100 nM)) and (**b**) relative current change of miRNA sequences and target HOTAIR lncRNA with respect to the NoT. Error bars represent the standard deviation across three separate experiments.

To confirm whether the capture-probe-HOTAIR target-specific interaction or a non-specific nucleotide interaction was responsible for generating the observed voltametric response, we investigated the specificity and the efficiency when the lncRNA biosensor was incubated with samples containing various potential interfering biomolecules (Figure 4). These biomolecules included non-complementary sequences miR-891 and miR-486. A very small decrease in current response was obtained for miR-891 and miR-486 (3.10 and 8.14 µA, respectively) compared to the no-template control. These data demonstrated a clear distinction between HOTAIR lncRNA targets and non-specific miR-891 and miR-486 (100 nM) (%RSD ≤ 5%, for *n* = 3), indicating that our assay is not significantly affected by non-specific miRNA binding present in the samples. However, the presence of perfectly matched HOTAIR lncRNA generated an approximately four-fold lower current response than that of the no target control (30.97 µA). These results indicate that the proposed biosensor shows great potential for clinical use due to its ideal specificity for detecting lncRNAs. To assess the reproducibility of the assay, three electrodes were prepared to detect lncRNA. The relative standard deviation of the measurements was less than 5% (*n* = 3) demonstrating an acceptable reproducibility of the proposed method for lncRNA detection. The main principle of detection among conventional nucleic acid biosensors relies on specific hybridization of target to complementary probes surface bound to a physical transducer/electrode. However, the major concern of such a two-dimensional capture approach is the interference of non-specific molecules. In contrast, our proposed method utilizes the selective capture of target HOTAIR lncRNA by complementary probes with subsequent lncRNA isolation via magnetic bead separation. More so, the magnetic washing and purification steps aid to eliminate matrix effects and substantially minimize non-specific interferences.

**Figure 5:**
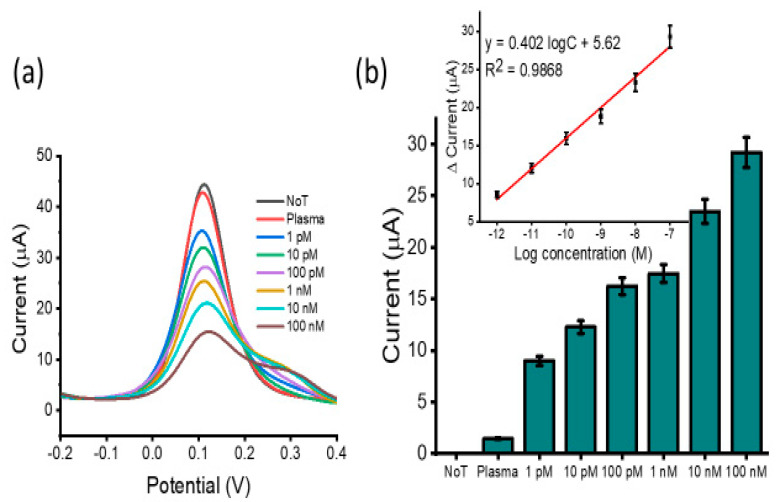
Assay testing in spiked plasma samples: (**a**) DPV readout displaying current output for serially diluted HOTAIR lncRNA in spiked plasma from 1 pM to 100 nM in reference to the NoT and (**b**) change in DPV current response for serially diluted HOTAIR targets in response to the NoT with linear calibration plots (inset). Error bars represent the standard deviation across three separate experiments.

Body fluids contain many species that may interfere with sensitive lncRNA detection. To investigate this possibility, DPV voltammograms were obtained for different concentrations of HOTAIR lncRNA in 10-fold diluted human plasma samples (1 pM to 100 nM). More so, to quantify HOTAIR lncRNA in patient samples, it is also critical to obtain an adequate detection range of the assay and the quantitative relationship between the electrochemical signal and the concentration of HOTAIR (Figure 5). Thus, the essential goal of this study was to create a discriminating lncRNA biosensor that could be used to analyze HOTAIR lncRNA directly in patient samples. The DPV responses had a strong linear relationship observed for the differing target HOTAIR concentrations, and in reference to the NoT, a greater change in current was observed with increasing target HOTAIR concentration. This demonstrated that the assay was able to detect HOTAIR lncRNA sequences in more challenging conditions. Furthermore, the assay’s dynamic range covered five orders of magnitude, facilitating the quantification of HOTAIR lncRNA in patients with various cancerous loads. The ability to detect specific targets of interest with high sensitivity is of great importance in various scientific settings where rapid analyte detection is essential [36]. The developed amplification-free platform was capable of detecting lncRNA to as low as 1 pM, which was comparable to a recently reported electrochemical biosensor based on Au NCs/MWCNT-NH_2_ nanostructure [35]. Our developed method’s LOD has a lower magnitude compared to the recently reported method based on duplex-specific nuclease-actuated cyclic enzymatic repairing-mediated signal amplification [37]. However, it is worth noting that we designed a low-cost and straightforward assay compared to several reported electrochemical approaches. The majority of these methods rely on coupling redox probes with another redox system to enhance the sensitivity.

**Figure 6:**
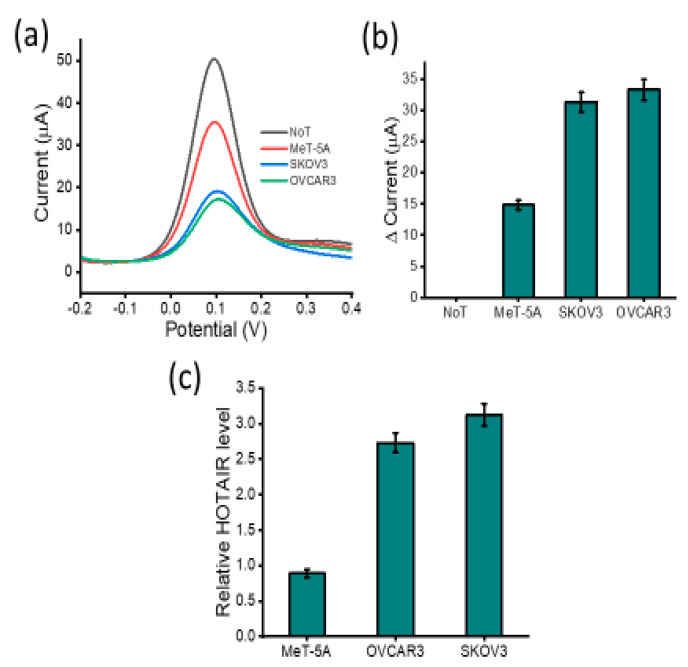
Assay cell line testing: (**a**) DPV readout displaying current output for benign (MeT-5A), carcinoma (OVCAR-3 and SKOV-3) in comparison to NoT and (**b**) change in DPV current response of the three cell lines in reference to the NoT. Error bars represent the standard deviation across three separate experiments. (**c**) RT-qPCR showing the relative HOTAIR expression levels in ovarian cancer cell line (SKOV3 and OVCAR3) and non-cancerous MeT-5A.

To determine whether our assay is applicable to real biological samples, our assay was tested with extracted total RNA from human ovarian cell lines (SKOV3 and OVCAR3) and one non-cancerous cell line (Met-5A) as a control (Figure 6). First, we used RT-qPCR to provide a quantitative comparison. Assay results demonstrate that HOTAIR lncRNA levels were expressed at differing levels across the different cell lines. Our assay demonstrated that HOTAIR lncRNA was more highly expressed in SKOV3 and OVCAR3 ovarian carcinoma cell lines than in the non-carcinoma MeT-5A cell lines as shown by the current responses. Increased levels of HOTAIR lncRNA detected correlated with the decreased current output as the sensor interface experienced coulombic repulsion from the approaching [Fe(CN)_6_]^3−/4−^ ions. These results were in good agreement with previously reported data, showing a similar profile for HOTAIR expression levels. The peak current of DPV correlated with the PCR bands (Figure 6c) from the ovarian cancer-specific cell lines (SKOV3 and OVCAR3) and a little higher DPV response from MeT-5A. Previous studies have reported the high expression of HOTAIR in ovarian cancer cells to have oncogenic effects, resulting in increased cell proliferation and growth properties [38]. Most importantly, our conventional RT-qPCR data support these findings and have noted approximately 3.0- and 2.5-fold higher HOTAIR expression in SKOV3 and OVCAR3 cell lines, respectively, compared to non-cancerous cell line MeT-5A. The reproducibility of DPV measurements for SKOV3 was derived to be an RSD of 4.82% (*n* = 3), while OVCAR3 was determined to be 4.13% (*n* = 3), MeT-5A was found to have an RSD of 4.98% (*n* = 3) and no target template was derived to have an RSD of 4.65% (*n* = 3). This indicates that our developed method can be used for accurate lncRNA biomarker detection in complex biological specimens such as blood and urine.

### 3.3. LncRNA Detection in Ovarian Cancer Patient Samples

**Figure 7:**
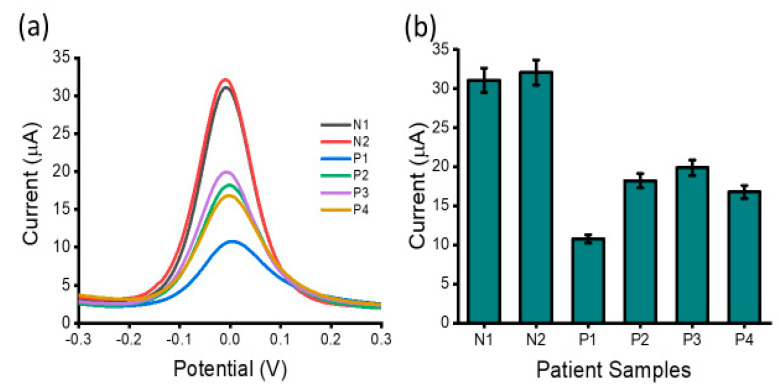
Detection of lncRNA in patient samples: (**a**) readout displaying current output for ovarian carcinoma negative patient samples (N1 and N2) and ovarian carcinoma positive patient samples (P1, P2, P3 and P4) and (**b**) carcinoma positive samples in comparison to carcinoma negative patient samples. Error bars represent the standard deviation across three separate experiments.

In addition to complex biological media testing with cell line samples, the practical feasibility of the proposed lncRNA biosensor for clinical application was investigated by detecting lncRNAs levels in extracted total RNA from four ovarian cancer high-grade serous sub-types (P1, P2, P3 and P4) and two benign samples (N1 and N2) (Figure 7). With the starting total RNA concentration of 10 ng µL^−1^, the DPV signals from the ovarian carcinoma samples were distinct from the benign samples showing high expression of the lncRNAs. The overexpression of HOTAIR lncRNA in ovarian cancer patients noted in the study is in agreement with previous reports which noted the association of HOTAIR expression levels with lymph node metastasis and a poor prognosis of patients with epithelial ovarian cancer [39]. The clinical data showed good inter-assay reproducibility as evidenced by the %RSD = 6%, (for *n* = 3). Thus, the established interfacial biosensing approach might become a potential tool for lncRNA assay in real samples.

### 3.4. Advantages and Disadvantages of the Assay

Our developed assay exhibits several distinct advantages for sensitive detection of HOTAIR such as the direct adsorption onto the electrode surface for electrochemical detection simplifies the assay procedure and avoiding any complex electrode premodification steps. The direct adsorption of HOTAIR target at the gold surface also enables faster assay time and reduced cost by eliminating the need for target amplification, target extension, labelling, and complex self-assembled monolayer surface chemistry. Introducing magnetic beads to directly capture and isolate the target lncRNAs from a complex biological specimen, this enables the elimination of the genomic DNA contamination often associated with conventional RNA assays and results in an extremely low background signal. In addition, our assay uses disposable screen-printed electrodes, which are inexpensive and avoid tedious cleaning of conventional disk electrodes, thereby providing a highly reproducible portable platform for lncRNA detection. Most importantly, the application of this assay is not limited to lncRNA detection; it could potentially be applied to many other clinically relevant nucleic acid biomarkers. The overall attribute of the developed sensor shows that it is compatible for integration with miniaturized, multiplexed, and decentralized analysis of RNA biomarkers with high translational potential. One of the major drawbacks limiting the sensitivity of our assay is non-specific adsorption. In addition, the folding tendency of lncRNAs into various secondary or tertiary structures when immobilized at the electrode surface reduces their structural stability. This issue decreases the analytical performance of the assay.

## 4. Conclusions

We reported a novel biosensor based on RNA–gold affinity interactions, for the early detection of ovarian cancer HOTAIR lncRNA via direct quantification with the [Fe(CN)_6_]^3−/4−^ redox couple. Our assay demonstrates good sensitivity with high specificity. Our assay shows great potential for success in the clinical setting, directly detecting lncRNA in high-grade serous sub-type plasma samples, with clear voltametric differentiation between carcinoma and benign plasma samples. Our method avoids the use of enzymes, antibodies, target amplification, or complex surface modification. The use of commercially available, cheap, and disposable gold screen-printed electrodes greatly reduces our assay time and cost. Our assay shows huge potential for commercialization in the diagnostic market due to its simple, inexpensive, easy to store and portable design, with huge potential for other nucleic-acid-based liquid biopsy biomarkers (e.g., cf DNA and ctDNA).

## Figures and Tables

**Table 1:** Sequences of DNA/ RNA nucleotides used in 5′ to 3′ orientation.

Oligonucleotides	5′-Sequences-3′
HOTAIR capture probe (Biotinylated)	ATC AAT TAA TTA GCG CCT CCC AGT CCC/3Bio 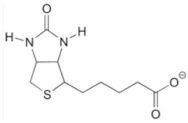
Synthetic HOTAIR lncRNA target	GGG ACU GGG AGG CGC UAA UUA AUU GAU UCC UUU GGA CUG UAA AAU AUG GCG GCG U
miR-891	UGC AAC GAA CCU GAG CCA CUG A
miR-486	UCC UGU ACU GAG CUG CCC CGA G
HOTAIR Forward primer sequence	AAG CAC CTC TAT CTC AGC CG
HOTAIR Reverse primer sequence	AGA ACC CTC TGA CAT TTG CCT
GAPDH Forward primer sequence	CCG GGA AAC TGT GGC GTG ATG G
GAPDH Reverse primer sequence	AGG TGG AGG AGT GGG TGT CGC TGT T

**Table 2:** Comparison of LODs.

Target	Techniques	Linear Range (M)	Detection Limit (M)	References
	DPV	10 pM–10 nM	0.247 pM	[29]
	DPV	1.0 pM–10 μM	0.886 pM	[30]
	DPV	10 pM–10 nM	2.57 pM	[31]
	DPV	100 fM–500.0 pM	84.3 fM	[32]
	DPV	100 fM–10 nM	50 fM	[33]
	I-T	1 fM–1.0 nM	1 fM	[34]
	DPV	10 fM–10.0 nM	42.8 fM	[35]
	DPV	1 pM–10 nM	1 pM	Present work

## Data Availability

Data are contained within this article or are available from the corresponding author upon reasonable request.

## References

[1] Mercer T.R., Dinger M.E., Mattick J.S. (2009). Long non-coding RNAs: Insights into functions. Nat. Rev. Genet..

[2] Yao R.-W., Wang Y., Chen L.-L. (2019). Cellular functions of long noncoding RNAs. Nat. Cell Biol..

[3] Bhan A., Mandal S.S. (2015). LncRNA HOTAIR: A master regulator of chromatin dynamics and cancer. Biochim. Biophys. Acta Rev. Cancer.

[4] Kopp F., Mendell J.T. (2018). Functional Classification and Experimental Dissection of Long Noncoding RNAs. Cell.

[5] Schmitt A.M., Chang H.Y. (2016). Long Noncoding RNAs in Cancer Pathways. Cancer Cell.

[6] Xue X., Yang Y.A., Zhang A., Fong K., Kim J., Song B., Li S., Zhao J.C., Yu J. (2016). LncRNA HOTAIR enhances ER signaling and confers tamoxifen resistance in breast cancer. Oncogene.

[7] Hajjari M., Salavaty A. (2015). HOTAIR: An oncogenic long non-coding RNA in different cancers. Cancer Biol. Med..

[8] Chen J., Deng B., Wu P., Li F., Li X.F., Le X.C., Zhang H., Hou X. (2016). Amplified binding-induced homogeneous assay through catalytic cycling of analyte for ultrasensitive protein detection. Chem. Commun..

[9] Lee T.-L., Xiao A., Rennert O.M. (2012). Identification of novel long noncoding RNA transcripts in male germ cells. Methods Mol. Biol..

[10] Lee C., Kikyo N. (2012). Strategies to identify long noncoding RNAs involved in gene regulation. Cell Biosci..

[11] Wang Z., Gerstein M., Snyder M. (2009). RNA-Seq: A revolutionary tool for transcriptomics. Nat. Rev. Genet..

[12] Koshiol J., Wang E., Zhao Y.D., Marincola F., Landi M.T. (2010). Strengths and Limitations of Laboratory Procedures for MicroRNA Detection. Cancer Epidemiol. Prev. Biomark..

[13] Islam M.N., Masud M.K., Haque M.H., Al Hossain M.S., Yamauchi Y., Nguyen N.-T., Shiddiky M.J.A. (2017). RNA Biomarkers: Diagnostic and Prognostic Potentials and Recent Developments of Electrochemical Biosensors. Small Methods.

[14] Soda N., Umer M., Kashaninejad N., Kasetsirikul S., Kline R., Salomon C., Nam-Trung N., Shiddiky M.J.A. (2020). PCR-Free Detection of Long Non-Coding HOTAIR RNA in Ovarian Cancer Cell Lines and Plasma Samples. Cancers.

[15] Islam M.N., Moriam S., Umer M., Phan H.-P., Salomon C., Kline R., Nguyen N.-T., Shiddiky M.J.A. (2018). Naked-eye and electrochemical detection of isothermally amplified HOTAIR long non-coding RNA. Analyst.

[16] Soda N., Rehm B.H.A., Sonar P., Nguyen N.-T., Shiddiky M.J.A. (2019). Advanced liquid biopsy technologies for circulating biomarker detection. J. Mater. Chem. B.

[17] Ahmed M., Carrascosa L.G., Sina A.A.I., Zarate E.M., Korbie D., Ru K.-L., Shiddiky M.J., Mainwaring P., Trau M. (2017). Detection of aberrant protein phosphorylation in cancer using direct gold-protein affinity interactions. Biosens. Bioelectron..

[18] Ibn Sina A.A., Koo K.M., Ahmed M., Carrascosa L.G., Trau M., Wandelt K. (2018). Interfacial Biosensing: Direct Biosensing of Biomolecules at the Bare Metal Interface. Encyclopedia of Interfacial Chemistry.

[19] Haque M.H., Gopalan V., Yadav S., Islam M.N., Eftekhari E., Li Q., Carrascosa L.G., Nguyen N.-T., Lam A.K., Shiddiky M.J.A. (2017). Detection of regional DNA methylation using DNA-graphene affinity interactions. Biosens. Bioelectron..

[20] Lu C.-H., Yang H.-H., Zhu C.-L., Chen X., Chen G.-N. (2009). A Graphene Platform for Sensing Biomolecules. Angew. Chem. Int. Ed..

[21] Song Y., Qu K., Zhao C., Ren J., Qu X. (2010). Graphene Oxide: Intrinsic Peroxidase Catalytic Activity and Its Application to Glucose Detection. Adv. Mater..

[22] Wu M., Kempaiah R., Huang P.-J.J., Maheshwari V., Liu J. (2011). Adsorption and Desorption of DNA on Graphene Oxide Studied by Fluorescently Labeled Oligonucleotides. Langmuir.

[23] Koo K.M., Sina A.A.I., Carrascosa L.G., Shiddiky M.J.A., Trau M. (2015). DNA–bare gold affinity interactions: Mechanism and applications in biosensing. Anal. Methods.

[24] Li H., Rothberg L. (2004). Colorimetric detection of DNA sequences based on electrostatic interactions with unmodified gold nanoparticles. Proc. Natl. Acad. Sci. USA.

[25] Islam T., Hasan M.M., Awal A., Nurunnabi M., Ahammad A.J.S. (2020). Metal Nanoparticles for Electrochemical Sensing: Progress and Challenges in the Clinical Transition of Point-of-Care Testing. Molecules.

[26] Masud M.K., Umer M., Hossain M.S.A., Yamauchi Y., Nguyen N.T., Shiddiky M.J.A. (2019). Nanoarchitecture Frameworks for Electrochemical miRNA Detection. Trends Biochem. Sci..

[27] Sina A.A.I., Howell S., Carrascosa L.G., Rauf S., Shiddiky M.J.A., Trau M. (2014). eMethylsorb: Electrochemical quantification of DNA methylation at CpG resolution using DNA–gold affinity interactions. Chem. Commun..

[28] Zhang J., Wang L., Pan D., Song S., Fan C. (2007). DNA hybridization “turns on” electrocatalysis at gold electrodes. Chem. Commun..

[29] Liu F., Xiang G., Jiang D., Zhang L., Chen X., Liu L., Luo F., Li Y., Liu C., Pu X. (2015). Ultrasensitive strategy based on PtPd nanodendrite/nano-flower-like@ GO signal amplification for the detection of long non-coding RNA. Biosens. Bioelectron..

[30] Liu F., Xiang G., Zhang L., Jiang D., Liu L., Li Y., Liu C., Pu X. (2015). A novel label free long non-coding RNA electrochemical biosensor based on green L-cysteine electrodeposition and Au–Rh hollow nanospheres as tags. RSC Adv..

[31] Yin H., Zhou Y., Yang Z., Guo Y., Wang X., Ai S., Zhang X. (2015). Electrochemical immunosensor for N6-methyladenosine RNA modification detection. Sens. Actuators B Chem..

[32] Rafiee-Pour H.-A., Behpour M., Keshavarz M. (2016). A novel label-free electrochemical miRNA biosensor using methylene blue as redox indicator: Application to breast cancer biomarker miRNA-21. Biosens. Bioelectron..

[33] Chen Y., Li Y., Yang Y., Wu F., Cao J., Bai L. (2017). A polyaniline-reduced graphene oxide nanocomposite as a redox nanoprobe in a voltammetric DNA biosensor for Mycobacterium tuberculosis. Microchim. Acta.

[34] Soda N., Umer M., Kasetsirikul S., Salomon C., Kline R., Nguyen N.-T., Rehm B.H.A., Shiddiky M.J.A. (2020). An amplification-free method for the detection of HOTAIR long non-coding RNA. Anal. Chim. Acta.

[35] Chen M., Wu D., Tu S., Yang C., Chen D., Xu Y. (2021). A novel biosensor for the ultrasensitive detection of the lncRNA biomarker MALAT1 in non-small cell lung cancer. Sci. Rep..

[36] Soda N., Clack K., Shiddiky M.J.A. (2022). Recent advances in liquid biopsy technologies for cancer biomarker detection. Sens. Diagn..

[37] Zhang Y., Wang X.-Y., Su X., Zhang C.-Y. (2019). Ultrasensitive detection of long non-coding RNAs based on duplex-specific nuclease-actuated cyclic enzymatic repairing-mediated signal amplification. Chem. Commun..

[38] Özeş A.R., Wang Y., Zong X., Fang F., Pilrose J., Nephew K.P. (2017). Therapeutic targeting using tumor specific peptides inhibits long non-coding RNA HOTAIR activity in ovarian and breast cancer. Sci. Rep..

[39] Qiu J.-J., Lin Y.-Y., Ye L.-C., Ding J.-X., Feng W.-W., Jin H.-Y., Zhang Y., Li Q., Hua K.-Q. (2014). Overexpression of long non-coding RNA HOTAIR predicts poor patient prognosis and promotes tumor metastasis in epithelial ovarian cancer. Gynecol. Oncol..

